# Opponent left-handedness does not affect fight outcomes for Ultimate Fighting Championship hall of famers

**DOI:** 10.3389/fpsyg.2014.00375

**Published:** 2014-04-30

**Authors:** Thomas V. Pollet, Bart R. Riegman

**Affiliations:** Department of Social and Organizational Psychology, VU University AmsterdamAmsterdam, Netherlands

**Keywords:** handedness, fighting hypothesis, laterality, mixed martial arts, combat sports

This paper is a commentary on Pollet et al. (2013), where both the incidence of left-handedness and its effect on outcomes were analyzed. One of the drawbacks is that these analyses were largely cross-sectional with only a small number of fights for any given fighter. Here, we explore the effects of left-handedness on fight outcomes in a small sample of elite UFC fighters with multiple fights from the same fighter.

All UFC fights by the current 10 UFC hall of fame fighters were coded by the second author B.R. (with assistance of Thomas V. Pollet) from a variety of online sour-ces (www.ufc.com; www.sherdog.com; www.mmajunkie.com; www.wikipedia.com) (*N* = 182 fights). The UFC hall of fame list consists of the most successful UFC fighters of all time and currently one non-fighter (UFC, 2014). For each fight we coded the outcome and handedness of the hall of famer and his opponent, if available. There were nine right-handed hall of famers and one left-hander (Royce Gracie). As there is only one left-hander we excluded his fights. Our analyses also exclude duplicate fights (i.e., hall of famers fighting one another). Central to our analyses is that we examine the effects of right-handers fighting left-handers, this is why we also exclude fights by Mark Coleman, who in our dataset, as a right-hander only fought right-handers. This leaves 75 fights with complete data on both fighters' handedness for analysis. The most straightforward test is a simple χ^2^ test for the 2 × 2 table. However, such a test does not take into account the nested structure of the data (i.e., fights belonging to the same hall of famer). Therefore, we also ran a Cochran-Mantel-Haenszel test (Agresti, 2002) and a Generalized Linear Mixed Model with binomial link (GLMM; McCulloch, 2006; Verbeke and Molenberghs, 2009), using the lme4 procedure in R 3.0.1 (R Development Core Team, 2008; Bates et al., 2012). If we find some indication for a fixed effect of opponent handedness in a GLMM, after accounting for the nested structure of the data (random intercept), then this suggests that the opponent's handedness significantly influences the outcome. We also test a model whereby we model a random intercept for the opponent, In order to account for multiple appearances of an opponent, as well as a random intercept for the hall of famer (see Hox, 2010). The lme4 package does not allow fitting quasibinomial models, we therefore also tested a GZLM with quasibinomial link, as over/underdispersion could affect the result from the GLMM. Given that our sample is (very) small we report one-tailed tests in favor of the hypothesis that left-handedness has an effect.

Hall of famers were not significantly less likely to win from left-handers (Figure 1; χ^2^ test = 0.17; Monte Carlo bootstrapped one-tailed *p*-value based on 10,000 samples = 0.39). The Cochran-Mantel-Haenszel test also does not support the conclusion that handedness affects outcomes in our sample (CMH test, one-tailed *p* = 0.44; estimate of common odds ratio = 0.813; 95%CI(odds ratio) = < 00.01 to 2.188). In the GLMM, with a random intercept for hall of famer, we found no evidence for a handedness effect (*B* = −0.214(±0.520); *Z* = −0.412, one-tailed *p* = 0.34). Accounting, for multiple appearances of the opponent or analyzing the data via a GZLM with quasi-binomial link does not alter this conclusion (respectively: *B* = −0.454(±0.628); *Z* = −0.723, one-tailed *p* = 0.235; *B* = −0.214(±0.527); *t* = −0.407, one-tailed *p* = 0.342).

**Figure 1:**
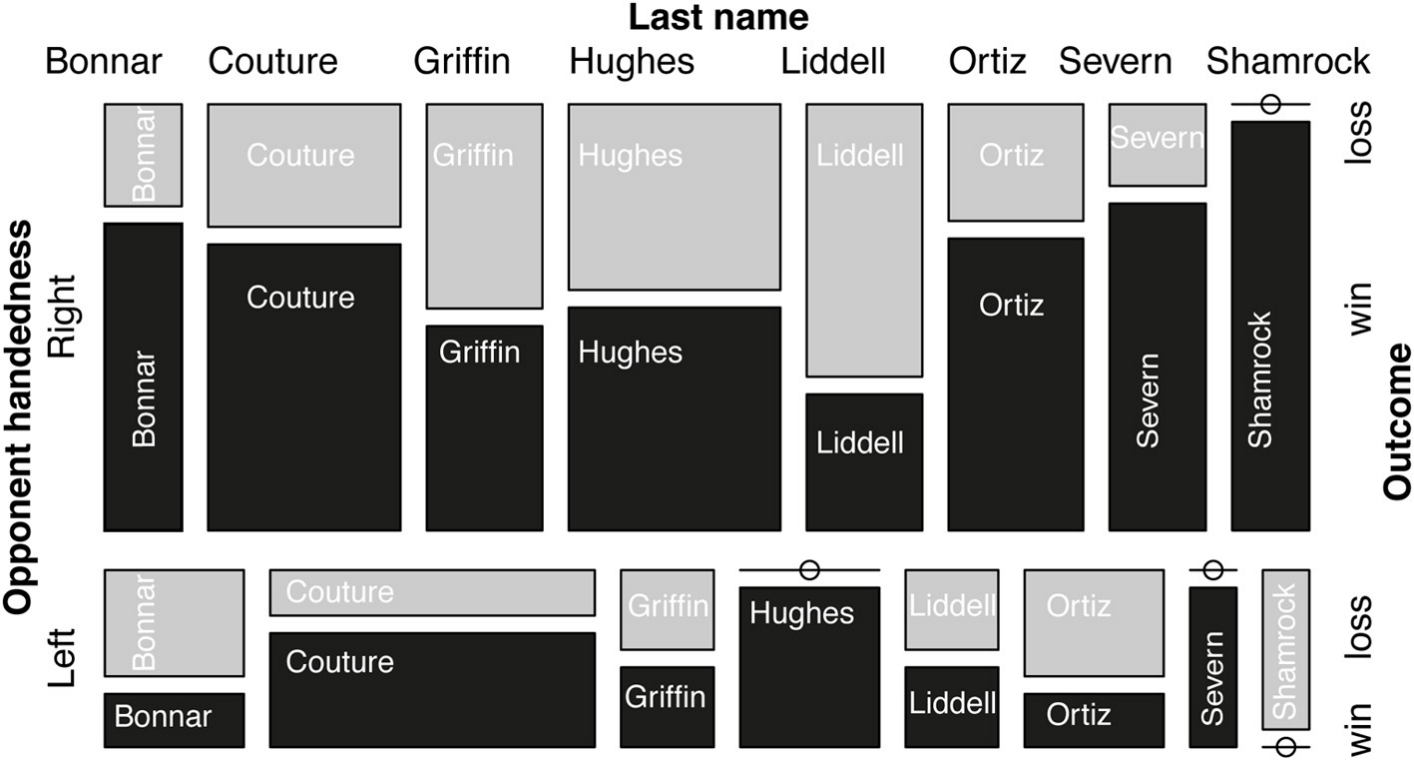
**A mosaic plot summarizing all the data (wins are in black, losses in gray; Ø = no data available in that category) (Meyer et al., 2006, 2013; Hofmann, 2008; Wickham and Hofmann, 2011)**.

Our analyses further support the conclusion that currently there is little evidence for an advantage of left-handers in mixed martial arts (MMA) based on wins. Across all tests there is little suggestion that Hall of Famers were more inclined to lose from left-handers. A much larger sample by Baker and Schorer (2013) also found that, while left-handers are overrepresented in MMA, there is no relationship between handedness and fight outcomes. One of the drawbacks of the Baker and Schorer (2013) study, however, is that Simpson's Paradox (Simpson, 1951) could not be ruled out (see Kievit et al., 2013). The pattern *between* fighters could be markedly different than that *within* fighters. While the win ratios analyzed by Baker and Schorer (2013) could be similar *between* left-handed and right-handed fighters, it is possible that the pattern *within* fighters is markedly different. It should also be noted that some evidence from other combat sports suggests that handedness does affect outcomes (e.g., *Wrestling*: Ziyagil et al., 2010; *Boxing*: Gursoy, 2009), it is possible that MMA is substantially different from these other sports or that the conclusion for MMA is premature.

This small additional study has many limitations, including a very small sample size and its nature (“the elite”). Nonetheless, our study further corroborates that there is little indication for an effect of handedness on *current* outcomes in MMA, unlike what the “fighting hypothesis” (Raymond et al., 1996; Llaurens et al., 2009; Faurie et al., 2011; Faurie and Raymond, 2013; also see: Schaafsma et al., 2012; Groothuis et al., 2013) would lead us to suggest. However, the fighting hypothesis does not necessarily have to imply that left-handers have better outcomes *currently* in the MMA. While there is no measurable effect on current outcomes in MMA, left-handers were found to be overrepresented in three studies [Pollet et al., 2013: 20.4% (*n* = 245); Baker and Schorer, 2013: 17.4% (*n* = 1486); Dochtermann et al., in preparation: 17% (*n* = 588)], as expected by the fighting hypothesis. This could imply that there is an equilibrium, whereby the benefit of left-handedness has allowed an influx of left-handers up until the point where the advantage has ceased. In order to establish whether this is plausible, further analyses are necessary (e.g., Dochtermann et al., in preparation), for example via longitudinal data on both amateurs and professionals. We therefore call for further research examining left-handedness in MMA to help understand why left-handers are overrepresented but, currently, not winning more fights. For now we conclude that, while left-handers are overrepresented in the UFC, and more broadly MMA, there is no substantial evidence that handedness reliably predicts fight outcomes.

## Conflict of interest statement

The authors declare that the research was conducted in the absence of any commercial or financial relationships that could be construed as a potential conflict of interest.
